# Increased Frequency of Low Back Pain in Recent Times: Does the Answer Lie in COVID-19?

**DOI:** 10.7759/cureus.50021

**Published:** 2023-12-06

**Authors:** Sreedhar Sathu, Ravi Kumar, Deepak K Maley, Srikanth Eppakayala, Adinarayana Kashyap, Akula NynaSindhu, Karra Madhu Latha, Maheshwar Lakkireddy

**Affiliations:** 1 Department of Orthopedics, All India Institute of Medical Sciences, Bibinagar, Hyderabad, IND; 2 Department of Orthopedics, All India Institute of Medical Sciences, Rajkot, Rajkot, IND; 3 Department of General Surgery, All India Institute of Medical Sciences, Bibinagar, Hyderabad, IND; 4 Department of Biochemistry, All India Institute of Medical Sciences, Bibinagar, Hyderabad, IND

**Keywords:** musculoskeletal system, vaccination, infection, covid-19, low back pain

## Abstract

Background

The COVID-19 pandemic has impacted many people's activities of daily living and health. It has also created economic burdens and caused mental turmoil across the world. Musculoskeletal symptoms, especially low back pain, have been observed in subjects of post-COVID-19 infection and post-vaccination.

Aim

In this study, we aimed to investigate the relationship between low back pain and COVID-19 infection and vaccination, as well as associated factors and characteristics.

Methods

We conducted a questionnaire-based cross-sectional observational study at All India Institute of Medical Science (AIIMS) Bibinagar between September 2021 and March 2022. We collected data from individuals through physical and Google Forms (Google, Mountain View, California).

Results

We included a total of 535 individuals in the study: 274 (51.2%) were previously positive for COVID-19 infection (group A), and 261 (48.8%) were vaccinated against COVID-19 without a history of COVID-19 infection (group B). Each group was divided into two categories based on whether they had low back pain before COVID-19 infection or vaccination. In group A, 90.1% of individuals experienced an aggravation of low back pain after COVID-19 infection, which was found to be significant (p<0.001). In group B, there was an insignificant increase in low back pain following COVID-19 vaccination (p=0.275). The study also revealed a significant association between comorbidities and low back pain in both groups (p<0.001). Additionally, several differences were observed between the two groups, including duration (p<0.001), severity (p=0.012), and intensity (p<0.001) of low back pain, usage of a back support or brace (p=0.043), and intake of vitamin D (p=0.002).

Conclusion

Low back pain is an ignored feature of one of the musculoskeletal symptoms of COVID-19 and was aggravated by COVID-19 infection in our patients compared to those who received the vaccination. The findings of this study have implications for raising awareness, improving management and rehabilitation, and guiding future research in this area.

## Introduction

COVID-19 infection had its origins in the Huanan seafood market of Southern China on December 29, 2019, with four index cases. Pneumonia of unknown etiology was detected in the city of Wuhan in China on December 31, 2019, and was subsequently reported to the World Health Organization (WHO). The coronavirus disease 2019 (COVID-19) outbreak was declared a pandemic on March 11, 2020 [1,2]. India reported an index case of COVID-19 in Kerala on January 30, 2020, a student who returned from Wuhan. The Ministry of Health and Family Welfare of the Indian Government declared community transmission on March 30, 2020 [3,4].

COVID-19 infection is caused by a novel human coronavirus named severe acute respiratory syndrome coronavirus 2 (SARS-CoV-2) by the International Committee on Taxonomy of Viruses. It is a new RNA virus strain that has not been identified in humans previously [5]. There is a substantial degree of homology in the genetic sequence and predicted viral human interaction between SARS-CoV-1 and SARS-CoV-2 strains [6,7]. In addition to direct infection of the cells, the airway's systemic inflammatory response to COVID-19 affects various organ systems, including the musculoskeletal system [8,9]. Most patients infected with SARS-CoV-2 have symptoms ranging from asymptomatic to mild or severe respiratory illness. SARS-CoV-2 resembles SARS-CoV-1, causing SARS and affecting multiple organ systems, including the musculoskeletal system. Myalgias, muscle dysfunction, osteoporosis, and osteonecrosis are common sequelae in patients with SARS infection, as revealed by epidemiological data of the 2002-2004 SARS pandemic [10]. Various studies of SARS-infected patients reported the musculoskeletal burden of the disease, including muscle, neurological, bone, and joint disorders [11,12]. On December 11, 2020, the Pfizer-BioNTech COVID-19 vaccine was approved for emergency use by the US Food and Drug Administration for those older than 16 years [13]. In India, the emergency use of Covishield (the name employed in India for the Oxford-AstraZeneca vaccine, which requires two doses recommended at a time interval of 12-16 weeks apart) and Covaxin, indigenously developed by Bharat Biotech in collaboration with the Indian Council of Medical Research and the National Institute of Virology to be given as two doses, 28 days apart. A free vaccination program was started in India on January 16, 2021 [14,15]. Studies advocated that inflammatory musculoskeletal symptoms may develop in close temporal association with COVID-19 vaccination despite a lack of a clear cause-effect relationship [16].

Mechanical/non-neurological low back pain is one of the most frequent and exorbitant illnesses of young people, leading to a significant loss of productivity. After six to eight weeks of management, 90% of those affected improve, with a recurrence of 60% in the following two years [17]. The economic burden of low back pain is also a big concern; in Western countries, it has been estimated to be 1-2% of the gross national product [18]. Of late, few research articles have been published on symptoms in post-COVID-19 patients. Specifically, only a few studies investigated the effect of COVID-19 on the musculoskeletal system in post-COVID-19 infected patients and post-vaccination populations [19-22].

We had a significant fraction of patients with low back pain after COVID-19 infection and post-vaccination subjects. We intended to investigate and establish/refute the relationship between COVID-19 infection and vaccination, and low back pain.

## Materials and methods

Study design

We conducted a cross-sectional observational study at All India Institute of Medical Sciences (AIIMS) Bibinagar between September 28, 2021, and March 24, 2022. The AIIMS institutional ethics committee approved the study (approval number AIIMS/BBN/IEC/SEP/2021/87-A) on September 9, 2021.

Data collection

We designed a questionnaire-based study with both physical forms and virtual Google Forms (Google, Mountain View, California). The questionnaire was written in English, and the scientific terms were written and explained in the local language. We verbally explained the study to eligible subjects attending the hospital's outpatient department. Once the participants consented to the study, we provided either physical or Google Forms (depending on comfort with technology) to collect the information. For the virtual form, we collected the phone numbers of the COVID-19 patients and vaccinated participants at AIIMS Bibinagar, and we sent Google forms after explaining the purpose of the study.

The questionnaire comprised four categories of questions. The first part of the questionnaire included demographic details, personnel habits, and co-morbidities. The second part included information about general well-being related to low back pain. The third part included COVID-19 infection data and their relationship with low back pain. The fourth part included COVID-19 vaccination-related data.

Study population

We screened patients admitted to the outpatient departments (OPDs) of AIIMS, Bibinagar, which included the post-covid clinic, orthopedic, and other OPDs.

Inclusion criteria

We included subjects who tested positive for COVID-19 infection or who were vaccinated with Covishield or Covaxin in the past without COVID-19 infection.

Exclusion criteria

We excluded subjects who were negative for COVID-19 infection and were not vaccinated against COVID-19, and those who were vaccinated with a history of COVID-19 infection. We also excluded subjects with neurological deficits or failed back syndrome.

Validity and reliability

The questionnaire was partly derived from other validated questionnaires available in the literature and modified for technical clarity and presentation [23,24]. The remaining part of the questionnaire was self-developed. We assessed the reliability of the questionnaire after obtaining responses to the self-administered questionnaire from respondents, including medical professionals of different broad and super-specialties. The entire set of questionnaire items was validated among a cluster (n=5) of medical experts working (interested) in the field. Each expert independently rated the relevance of each item using a four-point Likert scale (1 = not relevant, 2 = somewhat relevant, 3 = relevant, 4 = very relevant). Ratings of "3" and "4" were together considered a "favorable" response to the item, namely, the particular question was relevant; similarly, ratings of "1" and "2" were together considered an "unfavorable" response to the item, and the question was irrelevant. All the items were retained without major modifications, as the content validity index of the individual items was well above the cut-off, except for question number 2, which was deleted.

Study groups with sample size

We adopted a non-random convenience sampling technique for the study.

Sample size calculation

To calculate the sample size, we estimated a percentage or proportion in the case of the finite population. With a margin of error of 5%, a confidence level of 95%, and an available population size of 100,000, the minimum recommended sample size for the survey was estimated as 373 participants.

Ethical considerations

We began the study after obtaining prior approval from the Scientific Research Committee and Institutional Ethics Committee of AIIMS Bibinagar. The identity of the respondents was kept confidential.

Statistical analysis

We entered the data into a Microsoft Excel spreadsheet (Microsoft, Redmond, Washington), which was analyzed using SPSS 22 version (IBM Inc., Armonk, New York) software. We represented the categorical data in terms of frequencies and proportions. We used the chi-square test to determine the significance for qualitative data. We represented continuous data as means and standard deviations. We tested the normality of the continuous data with the Kolmogorov-Smirnov test and the Shapiro-Wilk test. We used the Student's independent t-test to determine significance and identify the mean difference between pairs of quantitative variables. A p-value (i.e., the probability that the result is true) of <0.05 was considered statistically significant after assuming all the rules of statistical tests.

## Results

**Figure 1:**
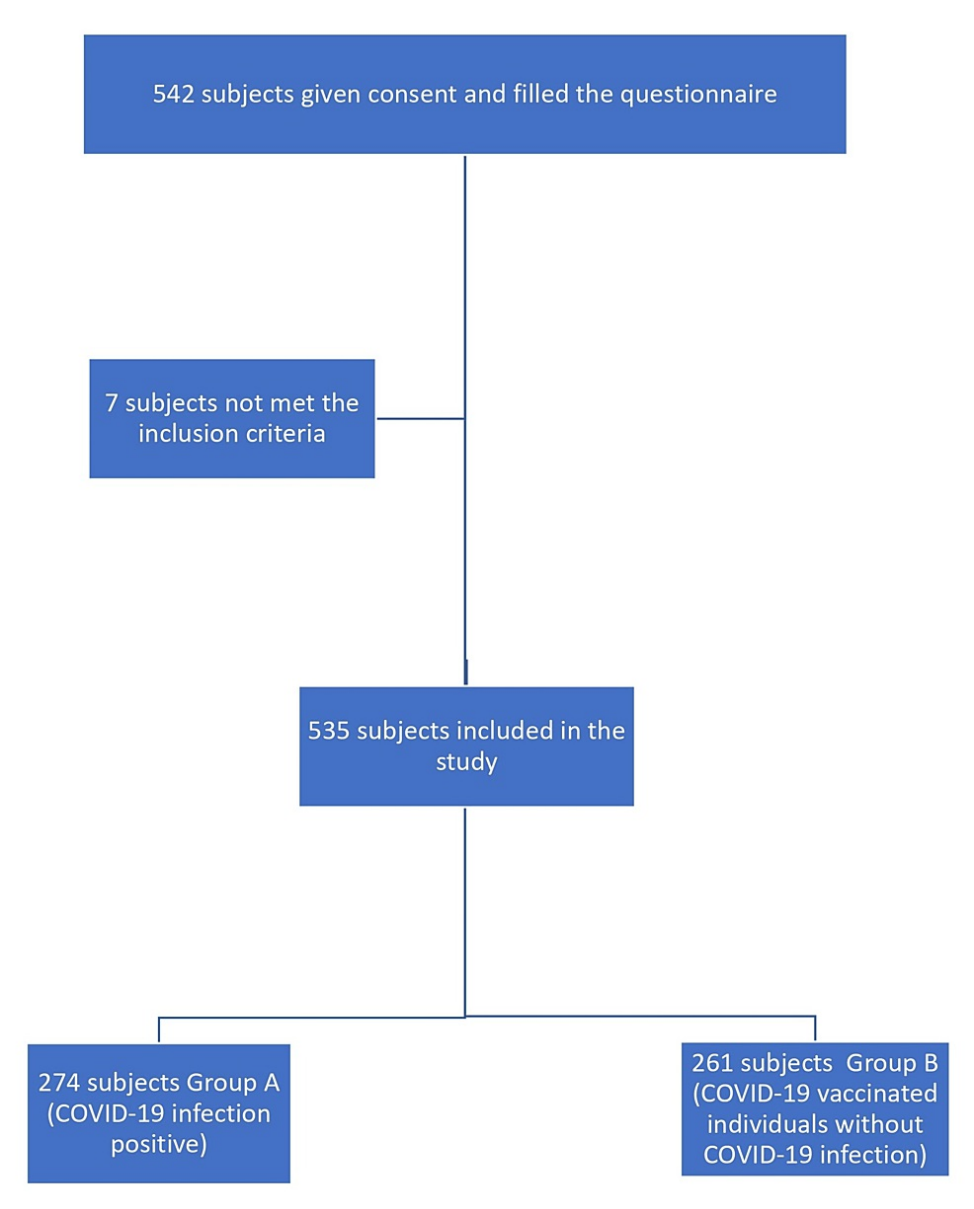
Flow chart showing the selection of study participants

In this study, 542 participants provided consent and filled out the COVID-19 questionnaire. However, seven participants did not meet the inclusion criteria. Out of 535 participants who met the inclusion and exclusion criteria, 274 participants had a history of COVID-19 infection (group A), and 261 participants were vaccinated against COVID-19 (group B); these vaccinated individuals were not infected with COVID-19 disease (Figure 1).

Demographic data

The ages of the participants ranged between 17 and 86 years, and most (78.87%, n=422) were between 21 and 50 years, with a mean age of 40.23 ± 12.25 years. Three hundred seventeen subjects (59.25%) were male, and 216 (40.37%) were female. Two participants preferred not to provide their gender. Thus, the male-to-female ratio was 1.47:1. The occupational data of the participants showed that 88 (16.45%) participants were healthcare workers, which included allopathic doctors, dental surgeons, veterinary doctors, nursing officers, physiotherapists, laboratory technicians, occupational therapy technicians, and X-ray technicians. Seventy-six (14.20%) subjects were housewives or homemakers, 60 (11.21%) were in service for the public and private sectors, 48 (8.97%) were business persons, 40 (7.48%) were agricultural workers, 23 (4.30%) were IT professionals, 18 (3.36%) were students, 11 (2.06%) were from the marketing field, 11 (2.06%) were drivers, 11 (2.06%) were daily laborers, 10 (1.87%) were retired employees, and the remaining were from various fields. One hundred fifteen (21.49%) participants did not provide their occupational details. Most of the participants in this study were from various places in the Telangana state, followed by Andhra Pradesh (Table 1).

**Table 1:** Demographic data of the study participants

Variable	Category	Frequency, n (%)
Age distribution (years)	17-20	6 (1.12%)
	21-30	125 (23.36%)
	31-40	184 (34.39%)
	41-50	113 (21.12%)
	51-60	75 (14.02%)
	61-70	24 (4.49%)
	71-80	6 (1.12%)
	81-86	2 (0.37%)
Gender	Male	317 (59.25%)
	Female	216 (40.37%)
	Prefer not to say	2 (0.37%)
Occupation of participant	Healthcare worker	88 (16.45%)
	Homemaker/housewife	76 (14.20%)
	Service in public/private sectors	60 (11.21%)
	Business person	48 (8.97%)
	Agriculture field	40 (7.48%)
	IT professional	23 (4.30%)
	Student	18 (3.36%)
	Marketing field	11 (2.06%)
	Driver	11 (2.06%)
	Daily laborer	11 (2.06%)
	Retired employee	10 (1.87%)
	Other occupations	24 (4.48%)
	Preferred not to mention	115 (21.49%)
Geographic location of the participants	Telangana	457 (85.42%)
	Andra Pradesh	53 (9.90%)
	Maharashtra	6 (1.12%)
	Tamil Nadu	5 (0.93%)
	Karnataka	3 (0.56%)
	Rajasthan	2 (0.37%)
	Delhi	2 (0.37%)
	Bihar	2 (0.37%)
	Uttar Pradesh	1 (0.19%)
	Himachal Pradesh	1 (0.19%)
	West Bengal	1 (0.19%)
	Odisha	1 (0.19%)
	London	1 (0.19%)

51.2% (n=274) of our participants were previously positive for COVID-19 infection (group A), and 48.8% (n=261) were vaccinated subjects with no history of COVID-19 infection (group B) (Table 2).

**Table 2:** Group categorization of study participants

	Number of participants (n=535)	Percentage (%)
COVID-19 positive (group A)	274	51.20%
Vaccinated without COVID-19 (group B)	261	48.80%

The mean age of the participants in groups A and B was 41.15 ± 12.32 years and 39.26 ± 12.11 years, and the mean body mass index (BMI) was 24.30 ± 4.08 and 24.16 ± 3.88, respectively. There was no significant difference in mean age and mean BMI between the two groups (Table 3).

**Table 3:** Age and BMI distribution comparison between groups A and B

	Group A (COVID-19 positive)		Group B (vaccinated without COVID-19)		p-value
	Mean	SD	Mean	SD	
Age (in years)	41.15	12.32	39.26	12.11	0.074
BMI	24.3	4.08	24.16	3.88	0.699

In groups A (COVID-19 positive) and B (vaccinated without COVID-19), 32.1% (n=88) and 49% (n=128) of participants were female, 67.2% (n=184) and 51% (n=133) were male, 47.4% (n=130) and 30.3% (n=79) consumed alcohol, and 32.1% (n=88) and 19.5% (n=51) had comorbidities, respectively. There was a significant difference in gender distribution (p<0.001), alcohol intake (p<0.001), and comorbidities (p=0.001) between groups A and B, whereas there was no significant difference in smoking (8.4%, n=23 and 5.7%,n=15) or BMI distribution (normal BMI of 67.5%, n=185 and 62.5%,n=163), respectively (Table 4).

**Table 4:** Sociodemographic profile comparison between groups A and B

		Group A (COVID-19 positive)		Group B (Vaccinated without COVID-19)		p-value
		Count (n)	Percentage (%)	Count (n)	Percentage (%)	
Gender	Female	88	32.10%	128	49.00%	<0.001*
	Male	184	67.20%	133	51.00%	
	Prefer not to say	2	0.70%	0	0.00%	
Alcohol intake	No	144	52.60%	182	69.70%	<0.001*
	Yes	130	47.40%	79	30.30%	
Smoking	No	251	91.60%	246	94.30%	0.233
	Yes	23	8.40%	15	5.70%	
Comorbidity	Yes	88	32.10%	51	19.50%	0.001^*^
	No	186	67.90%	210	80.50%	
BMI	<18.5	7	2.60%	9	3.40%	0.465
	18.5–24.9	185	67.50%	163	62.50%	
	25–29.9	57	20.80%	70	26.80%	
	30–34.9	17	6.20%	12	4.60%	
	>35	8	2.90%	7	2.70%	

There was a significant difference (p<0.001) in backache before COVID-19 between groups A and B at 81.4% (n=223) and 41% (n=107), respectively (Table 5).

**Table 5:** Backache before COVID-19 comparison between groups A and B χ2 =2.26, df =1, p<0.001* (Chi-squared test)

		Group A (COVID-19 positive)		Group B (Vaccinated without COVID-19)		Total	
		Number of participants (n)	Percentage (%)	Number of participants (n)	Percentage (%)	Number of participants (n)	Percentage (%)
Backache before COVID-19	Yes	223	81.40%	107	41.00%	330	61.70%
	No	51	18.60%	154	59.00%	205	38.30%
	Total	274	100.00%	261	100.00%	535	100.00%

In group A, out of 223 participants with backache before COVID-19, 90.1% (n=201) had aggravation of low back pain after COVID-19 infection, and 9.90% (n=22) had no aggravation. In 51 participants without low back pain before COVID-19, 100% had no low back pain after COVID-19 infection. There was a significant increase in low back pain after COVID-19 infection (Table 6).

**Table 6:** Comparison of backache before and after COVID-19 infection in group A χ2 =172.5, df =1, p<0.001* (Chi-squared test)

		Backache Before COVID-19					
		Yes		No		Total	
		Number of participants (n)	Percentage (%)	Number of participants (n)	Percentage (%)	Number of participants (n)	Percentage (%)
Backpain after COVID-19 infection	Aggravated	201	90.1%	0	0.0%	201	73.4%
	Not aggravated or absent	22	9.9%	51	100.0%	73	26.6%
	Total	223	100.0%	51	100.0%	274	100.0%

In group B, out of 107 participants with low back pain before COVID-19, 37.4% (n=40) had low back pain after COVID-19 vaccination, and out of 154 subjects without low back pain before COVID-19, 44.2% (n=68) had backache after vaccination. However, there was no significant difference in low back pain before COVID-19 and after vaccination (Table 7).

**Table 7:** Comparison of backache before and after COVID-19 vaccination in group B χ2 =1.194, df =1, p=0.275 (Chi-squared test)

		Backache before COVID-19					
		Yes		No		Total	
		Number of participants (n)	Percentage (%)	Number of participants (n)	Percentage (%)	Number of participants (n)	Percentage (%)
Backpain after COVID-19 vaccination	Backache present	40	37.4%	68	44.2%	108	41.4%
	Backache absent	67	62.6%	86	55.8%	153	58.6%
	Total	107	100.0%	154	100.0%	261	100.0%

In this study, there was no significant difference in gender, alcohol intake, smoking, or BMI between the two groups with low back pain. We observed a significant association between comorbidities and low back pain in the two groups. Of those in group A with low back pain, 35.4% (n=79) had comorbidities, whereas of those in group B with low back pain, 11.1% (n=12) had comorbidities (Table 8).

**Table 8:** Association between demographic profile and low back pain in groups A and B

		Group A (COVID-19 positive low back pain group) (n=223)		Group B (vaccinated without COVID-19 low back pain group) (n=108)		p-value
		Number of participants (n)	Percentage (%)	Number of participants (n)	Percentage (%)	
Gender	Female	79	35.40%	40	37.00%	0.759
	Male	143	64.10%	68	63.00%	
	Prefer not to say	1	0.40%	0	0.00%	
Alcohol intake	No	120	53.80%	63	58.30%	0.438
	Yes	103	46.20%	45	41.70%	
Smoking	No	207	92.80%	104	96.30%	0.214
	Yes	16	7.20%	4	3.70%	
Comorbidity	Yes	79	35.40%	12	11.10%	<0.001*
	No	144	64.60%	96	88.90%	
BMI	<18.5	6	2.70%	7	6.50%	0.071
	18.5-24.9	154	69.10%	80	74.10%	
	25-29.9	43	19.30%	19	17.60%	
	30-34.9	13	5.80%	1	0.90%	
	>35	7	3.10%	1	0.90%	

In group A with low back pain, the majority had backache over months (57%, n=127), moderate in severity (72.6%, n=162), and waxing and waning in intensity (80.7%, n=180); in the vaccinated low back pain group, the majority had backache over weeks (42.5%, n=17), moderate in severity (50%, n=20), and constant in intensity (72.5%, n=29). There was a significant difference in the duration (p<0.001), severity (p=0.012), and intensity (p<0.001) of backache between the two groups (Table 9).

**Table 9:** Association of low back pain characteristics between groups A and B

		Group A (COVID-19 positive low back pain group) (n=223)		Group B (vaccinated without COVID-19 low back pain group) (n=40)		Total		p-value
		Number of participants (n)	Percentage (%)	Number of participants (n)	Percentage (%)	Number of participants (n)	Percentage (%)	
How long have you been suffering from backache?	Over days	11	4.90%	12	30.00%	23	8.70%	<0.001*
	Over weeks	49	22.00%	17	42.50%	66	25.10%	
	Over months	127	57.00%	11	27.50%	138	52.50%	
	Over years	36	16.10%	0	0.00%	36	13.70%	
Severity of backache	Mild	25	11.20%	11	27.50%	36	13.70%	0.012^*^
	Moderate	162	72.60%	20	50.00%	182	69.20%	
	Severe	33	14.80%	9	22.50%	42	16.00%	
	Excruciating	3	1.30%	0	0.00%	3	1.10%	
How does the backache vary?	Constant	40	17.90%	29	72.50%	69	26.20%	<0.001*
	Increases on bending	1	0.40%	0	0.00%	1	0.40%	
	Rarely	1	0.40%	0	0.00%	1	0.40%	
	Standing continuously for 30 minutes	1	0.40%	0	0.00%	1	0.40%	
	Waxing and waning	180	80.70%	11	27.50%	191	72.60%	

There was no significant difference in disturbed sleep, consultation of a doctor, medication for backache, injections to the spine, surgery for backache, congenital (birth) or developmental (growth) defect, infectious or inflammatory pathology of the spine, history of significant trauma to the spine, neoplastic/ tumorous spine pathology, diagnosis of a degenerative/age-related spine disorder, physiotherapy for backache, or back-strengthening exercises between the two groups. There was a significant difference in the investigation for backache (p=0.002), ever using a back support/brace (p=0.043), and vitamin D supplementation (p=0.002) between the two groups. Group A with low backache had higher rates of being investigated for backache, using a back support/brace, and vitamin D supplementation, compared with group B low back pain (Table 10).

**Table 10:** Low back pain affecting routine activities among the subjects within groups A and B

		Group A (COVID-19 with low back pain group) (n=223)		Group B (Vaccinated without COVID-19 low back pain group) (n=40)		Total		p-value
		Number of participants (n)	Percentage (%)	Number of participants (n)	Percentage (%)	Number of participants (n)	Percentage (%)	
Sleep disturbed	No	190	85.20%	35	87.50%	225	85.60%	0.703
	Yes	33	14.80%	5	12.50%	38	14.40%	
Consulted a doctor for backache	No	73	32.70%	12	30.00%	85	32.30%	0.733
	Yes	150	67.30%	28	70.00%	178	67.70%	
Investigated for backache	No	96	43.00%	28	70.00%	124	47.10%	0.002*
	Yes	127	57.00%	12	30.00%	139	52.90%	
Used any medication for backache	No	39	17.50%	5	12.50%	44	16.70%	0.436
	Yes	184	82.50%	35	87.50%	219	83.30%	
Injections to spine for backache	No	216	96.90%	39	97.50%	255	97.00%	0.828
	Yes	7	3.10%	1	2.50%	8	3.00%	
Ever suggested to go for surgery for backache	No	212	95.10%	40	100.00%	252	95.80%	0.151
	Yes	11	4.90%	0	0.00%	11	4.20%	
Ever used a back support/ brace	No	202	90.60%	40	100.00%	242	92.00%	0.043*
	Yes	21	9.40%	0	0.00%	21	8.00%	
Vitamin D supplementation	No	86	38.60%	26	65.00%	112	42.60%	0.002*
	Yes	137	61.40%	14	35.00%	151	57.40%	
Congenital (Birth defect) or Developmental (Growth defect) abnormality of spine	I don’t know	4	1.80%	0	0.00%	4	1.50%	0.393
	No	219	98.20%	40	100.00%	259	98.50%	
Suffered any infectious or inflammatory pathology of spine	I don’t know	5	2.20%	0	0.00%	5	1.90%	0.577
	No	217	97.30%	40	100.00%	257	97.70%	
	Yes	1	0.40%	0	0.00%	1	0.40%	
Any history of significant trauma to spine	I don’t know	2	0.90%	0	0.00%	2	0.80%	0.577
	No	217	97.30%	40	100.00%	257	97.70%	
	Yes	4	1.80%	0	0.00%	4	1.50%	
History of neoplastic/ tumorous Spine pathology	I don’t know	3	1.30%	0	0.00%	3	1.10%	0.695
	No	219	98.20%	40	100.00%	259	98.50%	
	Yes	1	0.40%	0	0.00%	1	0.40%	
Diagnosed with a degenerative/ age-related spine disorder	I don’t know	5	2.20%	0	0.00%	5	1.90%	0.339
	No	218	97.80%	40	100.00%	258	98.10%	
Physiotherapy for backache	No	199	89.20%	39	97.50%	238	90.50%	0.101
	Yes	24	10.80%	1	2.50%	25	9.50%	
Back-strengthening exercises	No	172	77.10%	33	82.50%	205	77.90%	0.658
	Not anymore	4	1.80%	1	2.50%	5	1.90%	
	Yes	47	21.10%	6	15.00%	53	20.20%	

## Discussion

In India, the first case of COVID-19 infection was detected on January 30, 2021, and WHO declared COVID-19 to be a pandemic, followed by a community transmission declaration in India [25]. Apart from respiratory symptoms ranging from mild to moderate to severe in intensity, COVID-19 affects various other organ systems and exhibits a variety of manifestations, including musculoskeletal symptoms [26]. Despite musculoskeletal manifestations being common in the early phase of COVID-19 infection for various reasons, it has been underreported because of disease severity in other organ systems [27]. In a study by Bakilan et al. [28], fatigue was reported in 72% of post-acute COVID-19 patients, low back pain was reported in 71% of patients, and myalgias and arthralgias were reported in 61% and 44% of patients, respectively. The angiotensin-converting enzyme 2 (ACE2) receptors, which are homologous in configuration and genetic makeup, are targets of both SARS-CoV-1 and SARS-CoV-2. Although the precise mechanism is unclear due to the expression of ACE2 receptors in skeletal muscle, adipocytes, and endothelial cells within the musculoskeletal system, they are target sites for SARS-CoV-2 infection, which might also explain the musculoskeletal manifestations of the disease. The principal pathophysiology of musculoskeletal manifestations related to COVID-19 includes cytokine storm, high expression of IL-6 and other proinflammatory mediators, development of a prothrombotic state, and autoimmunity [28-31]. In this study, we aimed to determine the frequency of low back pain of post-COVID-19 positive individuals (group A) and post-vaccination individuals without COVID-19 infection (group B). We also aimed to compare the analyzed demographic factors associated with low back pain and to evaluate the characteristics of low back pain in these two groups.

To the best of our knowledge, this is the only study conducted to determine low back pain related to COVID-19 in chronic settings, including COVID-19 positive and COVID-19 vaccination subjects. The ages of our 535 participants ranged from 17 to 86 years, and 78.87% (n=422) were 21 to 50 years of age, with a mean age of 40.23 ± 12.25 years. 59.25% (n=317) of our participants were male. Most of the study participants were from a single geographical area. Having divided the participant data into two groups, 51.2% (n=274) were categorized as group A (COVID-19 infection positive), and 48.8% (n=261) were categorized as group B (vaccinated group without COVID-19 infection), with mean ages of 41.15 ± 12.32 years and 39.26 ± 12.11 years, and mean BMIs of 24.30 ± 4.08 and 24.16 ± 3.88, respectively. Cipollaro et al. [32] analyzed data from retrospective single-center studies in 12,046 COVID-19 patients and reported various musculoskeletal manifestations and epidemiological characteristics. The average age of the patients was 52.13 years; 54% were male, and 46% were female. Cipollaro et al. observed that myalgia, arthralgia, and fatigue symptoms were continuously present from the initial stage to the most severe stage of the COVID-19 disease. Our study differed from that of Cipollaro et al., as we analyzed the post-COVID-19 symptoms and compared them with those with pre-COVID-19 status. In group A (COVID-19 infection positive), 223 individuals had backache before COVID-19 infection, in which 90.1% (n=201) had experienced the aggravation of their low back pain after COVID-19 infection, and 9.90% (n=22) of individuals had no aggravation of their low back pain after infection. Our statistical analysis suggested that there was a significant increase in low back pain after COVID-19 infection. A systematic review and meta-analysis by Abdullahi et al. [33] included 11,069 subjects, of which 5168 were male, and the mean age ranged from 24 to 95 years. They reported that the pooled prevalence of low back pain in COVID-19 patients was 10% (95% confidence interval: 0.01-0.23). However, in that study, 2377 patients were in critically ill condition. Uz et al. [34] conducted a study on 99 COVID-19 polymerase chain reaction-positive inpatients. The mean age of the patients (53 male and 46 female) was 48.80 ± 14.64 years, and 59 (60%) patients had at least one comorbidity. Low back pain was observed in 50.5% (n=50) of the patients. Jena et al. [35] conducted a study on 182 hospitalized COVID-19 patients and reported low back pain in 22.53% (n=41) of patients.

In our study, out of 107 participants with low back pain before COVID-19 in group B, 37.4% (n=40) had low back pain after COVID-19 vaccination. Out of 154 participants without low back pain before COVID-19, 44.2% (n=68) had backache after vaccination, which suggested that there was no significant difference in low back pain before COVID-19 and after vaccination. In comparison with group B (vaccinated individuals), group A (COVID-19 infection) had significantly higher levels of low back pain. However, there was a significant difference in pre-COVID-19 and pre-vaccination low back pain status, gender distribution, and alcohol intake between the groups.

In our study, we observed a significant association (p<0.001) between comorbidities (e.g., hypertension, diabetes mellitus, and hypothyroidism) and low back pain in the two groups. 35.4% (n=79) had comorbidities in the COVID-19-positive (group A) low back pain group, whereas 11.1% (n=12) had comorbidities among the vaccinated low back pain group (group B). The COVID-19 patients with comorbidities had a higher chance of developing a severe progression of the disease. Low back pain has been associated with the presence and severity of COVID-19 [36]. In our study, with respect to low back pain duration (p<0.001), severity (p=0.012), and intensity (p<0.001), we observed a significant difference between the two groups. In the COVID-19 infection low back pain group, 57.0% (n=127) had low back pain over the course of months, whereas in the vaccinated low back pain group, 42.5% (n=17) had it over the course of weeks. In both the COVID-19 infected and the vaccinated low back pain groups, the majority of patients had moderate-type low back pain (72.6%,n=162 and 50%,n=20 respectively); 80.7% (n=180) had intermittent-type low back pain in the COVID-19 infection low back pain group, and 72.5% (n=29) had constant-type low back pain in the vaccinated low back pain group. In both groups, 57.0% (n=127) and 30.0% (n=12) were investigated for low back pain (p=0.002); 9.4% (n=21) and 0% used brace support (p=0.043); and 61.4% (n=137) and 35.0% (n=14) had taken vitamin D3 supplementation (p=0.002), respectively. Therefore, significant differences were observed between the groups.

In post-COVID-19 patients, we must give significance to complaints of low back pain because it can be due to intramuscular edema in multifidus and erector spinae, and it may be associated with raised c-reactive protein, erythrocyte sedimentation rate, creatinine kinase, and D-dimer levels. These features of paraspinal myositis can be obvious in magnetic resonance imaging and may be absent in the dorsal and cervical regions. Along with muscular viral load, an immune-mediated para-infectious inflammatory response, adverse effects of drugs, and critical illness related to myopathy could be causes of low back pain [37,38].

Our study had certain limitations, such as recall and selection bias. The data of low back pain and characteristics in the vaccinated low back pain group were significantly less compared with the COVID-19-infected low back pain group. We did not include the association between low back pain characteristics and disease characteristics in the study. We recommend further studies with a larger sample size to validate the results of our research and arrive at a more precise estimate of predicting the low back pain manifestation and its characteristics in the COVID-19 infected and vaccinated population to create a structured follow-up protocol for its effective management.

## Conclusions

Our results showed that low back pain was more common in post-COVID-19 and post-vaccination individuals. Low back pain was much more aggravated in those who had low back pain previously. However, when compared with vaccinated individuals, infected individuals were affected more. We observed a significant association between comorbidities (e.g., hypertension, diabetes mellitus, and hypothyroidism) and low back pain. The duration of low back pain was also longer in the infected group. Therefore, orthopedic doctors should determine a history of exposure to COVID-19 in cases of low back pain as a matter of routine. This study provides a foundation to assess low back pain in post-COVID-19 and vaccinated individuals and its associated factors. Despite the limitations of this study, our results should hopefully inspire others within the scientific community to conduct further research on low back pain as a symptom of long COVID in post-COVID-19 survivors.
